# Transition shock and perceived patient safety culture among newly graduated nurses: a latent profile and chained mediation analysis

**DOI:** 10.3389/fmed.2026.1841000

**Published:** 2026-06-29

**Authors:** Ying Lu, Xiangjin Diao, Jiajia Yang, YongHui Wan, Shuai Cao

**Affiliations:** 1 Department of Cardiology, Institute of Cardiovascular Diseases, Xiangyang Central Hospital, Affiliated Hospital of Hubei University of Arts and Science, Xiangyang, China; 2 Department of Nursing, Xiangyang Central Hospital, Affiliated Hospital of Hubei University of Arts and Science, Xiangyang, China; 3 Department of Nursing, Chongqing Health Center for Women and Children, Chongqing, China; 4 Department of Nursing, Renmin Hospital of Wuhan University, Wuhan, China

**Keywords:** latent profile analysis, mediation analysis, newly graduated registered nurses, patient safety culture, transition shock

## Abstract

**Background:**

Newly graduated registered nurses (NGRNs) are particularly vulnerable to transition shock (TS), which may adversely affect perceived patient safety culture (PSC). However, the heterogeneity of TS and the psychological mechanisms through which it influences PSC remain insufficiently understood.

**Aim:**

To explore latent profiles of TS among NGRNs and to examine their associations with perceived PSC, with a focus on the mediating roles of psychological capital (PsyCap) and second victim experience (SVE), in order to inform targeted interventions for supporting NGRNs and enhancing PSC.

**Methods:**

A cross-sectional study was conducted among 438 NGRNs from 18 public hospitals in China. Latent profile analysis (LPA) was used to identify distinct TS profiles. Differences in PSC across profiles were examined. Mediation analysis was performed to explore the roles of PsyCap and SVE.

**Results:**

Three TS profiles were identified: low TS–social type (36.5%), moderate TS–competence type (48.0%), and high TS–psychological type (15.5%). PSC differed significantly across profiles (*p* < 0.001), with higher TS associated with lower PSC. PsyCap significantly mediated the relationship between TS and PSC across all profiles. SVE showed a significant mediating effect in the low and moderate TS groups but not in the high TS group. A significant chain mediation pathway was observed in the low and moderate TS profiles.

**Conclusion:**

TS among NGRNs is heterogeneous and negatively associated with PSC. PsyCap and SVE play important mediating roles, highlighting the combined effects of psychological resource depletion and negative professional experiences. These findings provide evidence for targeted interventions to support NGRNs and PSC.

## 1 Introduction

Patient safety remains a key concern in the healthcare sector (1) Statistics indicate that one in every 300 patients dies as a result of preventable medical harm, while approximately 40% of patients experience harm during treatment, of which up to 80% may be avoidable (2, 3). As the largest professional group in healthcare, nurses are recognized as playing a crucial role in ensuring patient safety and delivering high-quality care (4). Therefore, enhancing nurses' perceived patient safety culture (PSC) and promoting safe practices have become key priorities in both nursing management and education.

Newly graduated registered nurses (NGRNs) require particular attention in the context of patient safety management. NGRNs are typically defined as nurses within the first 2 years of clinical practice following graduation (5). Compared with experienced nurses, they often have limited clinical experience and are more likely to experience anxiety, hesitation, and judgment errors when confronted with complex patient conditions, emergencies, and multitasking demands (6). As a result, they are considered a high-risk group for adverse nursing events. Previous research (7) has shown that NGRNs account for 61.4% of such events. In addition, difficulties in role adaptation, high work-related stress, and limited clinical competence frequently contribute to negative professional experiences, which may affect both workforce stability (8, 9) and patient safety.

The transition from student to professional nurse is widely recognized as complex and challenging. During this process, NGRNs often experience transition shock (TS) (10, 11), which is defined as confusion, uncertainty, and doubt arising from changes in roles, relationships, knowledge, and responsibilitie (12). High levels of TS have been associated with negative emotional states, such as anxiety and tension, as well as physical symptoms, including fatigue and insomnia (11). These challenges may lead to reduced work performance (13) and increased turnover intention (10). However, most existing studies (6, 14, 15) have treated NGRNs as a homogeneous group, with limited attention given to individual differences in TS experiences.

To better capture individual differences in TS, latent profile analysis (LPA) has been proposed as a person-centered approach that identifies subgroups based on response patterns across multiple variables (16). Recent nursing studies have increasingly applied LPA to identify heterogeneous psychological characteristics among nurses, such as distinct psychological capital profiles, demonstrating its value for informing tailored management and intervention strategies (17). This method enables a more nuanced understanding of TS heterogeneity and provides a basis for targeted interventions. However, research applying LPA to TS among NGRNs remains scarce, particularly in relation to perceived PSC.

The concept of the second victim (SV) refers to healthcare professionals who experience psychological or emotional distress following involvement in adverse events (18). Due to limited experience and high stress, NGRNs may be more vulnerable to both adverse events and becoming second victims (19). A recent study (20) showed that NGRNs who experience patient safety incidents frequently report substantial second victim distress and often perceive insufficient support following such events, highlighting their vulnerability during the transition period. Following such events, they may experience guilt, anxiety, insomnia, self-doubt, and reduced self-efficacy, collectively referred to as second victim experience (SVE) (21). Without adequate support, these reactions may persist and develop into burnout or psychological distress, ultimately exerting negative effects on both safety behaviors and perceived PSC (21, 22).

Psychological capital (PsyCap) is conceptualized as a key positive psychological resource encompassing self-efficacy, hope, optimism, and resilience (23). Higher PsyCap enables individuals to maintain positive expectations under stress and to adopt proactive coping strategies (24). A meta-analysis (25) indicated that nurses' PsyCap levels have declined since 2019, with NGRNs exhibiting the lowest levels. For NGRNs, PsyCap not only facilitates adaptation to clinical challenges but may also shape perceptions of the work environment and patient safety culture (26). Conversely, high levels of TS may deplete PsyCap, resulting in reduced confidence, diminished optimism, and more negative perceptions of PSC. From the perspective of conservation of resources (COR) theory (27), stress is experienced when psychological resources are threatened or depleted, providing a useful framework for understanding how TS may influence PsyCap and subsequent outcomes.

In addition, PsyCap and SVE may operate as interconnected mechanisms rather than as independent factors. High levels of TS may initially deplete positive psychological resources, thereby reducing resilience and self-efficacy (28), which in turn may increase vulnerability to SVE when facing adverse events or high-pressure situations (29). This suggests that TS may influence PSC not only directly but also indirectly through a sequential pathway in which reduced PsyCap increases susceptibility to SVE. However, empirical evidence supporting this chain mediation mechanism remains limited, especially among NGRNs.

Based on these gaps, this study aimed to identify latent profiles of TS among NGRNs using LPA, to compare differences in perceived PSC across profiles, and to examine the mediating roles of PsyCap and SVE in the relationship between TS and PSC. The findings are expected to provide a clearer understanding of the underlying mechanisms and to inform targeted interventions to support NGRNs. According to COR theory, individuals strive to obtain, maintain, and protect valuable resources, while resource loss is more salient than resource gain (30). In the context of the transition to professional practice, TS may represent a significant source of resource depletion. PsyCap can be viewed as an important personal resource that enables NGRNs to cope with workplace stressors, whereas SVE may reflect further resource loss following adverse clinical events. Therefore, COR theory provides a useful framework for understanding how TS may influence perceptions of PSC through PsyCap and SVE. Based on the theoretical framework, the following hypotheses are proposed:

- **Hypothesis 1:** TS among NGRNs is heterogeneous, and distinct latent profiles can be identified.
- **Hypothesis 2:** Different TS profiles are associated with varying levels of perceived PSC, with higher TS associated with lower PSC.
- **Hypothesis 3:** PsyCap mediates the relationship between TS and PSC.
- **Hypothesis 4:** SVE mediates the relationship between TS and PSC.
- **Hypothesis 5:** PsyCap and SVE exert a chain mediating effect in the relationship between TS and PSC.

## 2 Methods

### 2.1 Study design and participants

This cross-sectional study was conducted between January and February 2026 in 18 public hospitals in China and was reported in accordance with the STROBE checklist. The participants were NGRNs. Inclusion criteria were as follows: (1) possession of a valid registered nurse qualification; (2) 6 months to 2 years of clinical experience; and (3) provision of informed consent. Exclusion criteria were as follows: (1) prior nursing work experience; and (2) incomplete questionnaires or responses exhibiting obvious response patterns.

### 2.2 Sample size

**Figure 1:**
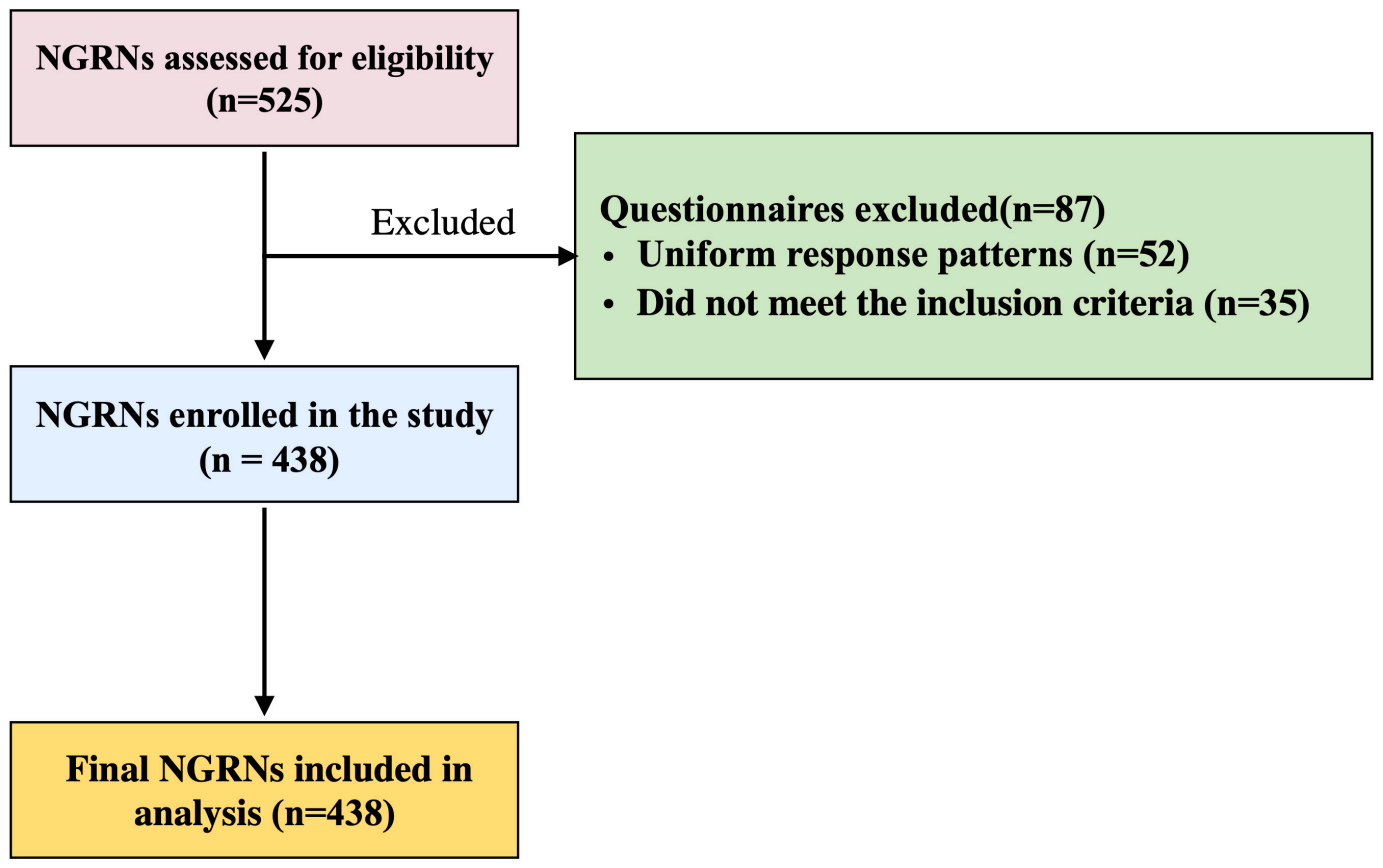
Flow diagram of participant inclusion and exclusion process.

This study utilized G^*^Power 3.1 software for sample size estimation (31). Based on a medium effect size (*f*^2^ = 0.15), a significance level of α = 0.05, and a statistical power of 0.90, the minimum required sample size was estimated to be 212 participants. After allowing for a 15% invalid response rate, the target sample size was 249 participants. A total of 438 NGRNs were ultimately included in this study, substantially exceeding the minimum sample size requirement and providing adequate support for the planned statistical analyses. The participant selection process is illustrated in Figure 1.

### 2.3 Instruments

#### 2.3.1 Demographic information

A self-designed questionnaire was used to collect demographic data, including age, gender, education level, hospital grade, employment type, department, and number of night shifts.

#### 2.3.2 Safety attitudes questionnaire (SAQ)

Perceived PSC was measured using the Safety Attitudes Questionnaire (SAQ), which was adapted for the Chinese context by Chen (32) based on Sexton's (33) original scale. The scale includes 24 items across five dimensions: teamwork climate (7 items), job satisfaction (6 items), stress recognition (4 items), unit safety climate (4 items), and perception of management (3 items). Items were rated on a 5-point Likert scale (1 = strongly disagree to 5 = strongly agree), with higher scores indicating more positive PSC. The scale demonstrates good reliability and validity, with a Cronbach's α of 0.886; in this study, the Cronbach's α for the scale was 0.944.

#### 2.3.3 The second victim experience and support tool (SVEST)

SVE was assessed using the SVEST, adapted for the Chinese context by Chen (34), based on Burlison's (35) original scale. The scale includes 24 items across six dimensions: psychological distress, physical distress, professional distress, colleague support, managerial support, and non-work-related support. Items are rated on a 5-point Likert scale. Distress dimensions are positively scored, while support dimensions are reverse scored; higher scores indicate more severe SVE and less support. The scale demonstrates good reliability and validity, with a Cronbach's α of 0.824; in this study, the Cronbach's α for the scale was 0.840.

#### 2.3.4 Psychological capital questionnaire (PCQ)

PsyCap was measured using the Psychological Capital Questionnaire (PCQ), adapted for the Chinese context by Luo (36), based on Luthans' (37) work. The questionnaire consists of 20 items across four dimensions: self-efficacy (6 items), hope (6 items), resilience (5 items), and optimism (3 items). Items are rated on a 6-point Likert scale (1 = strongly disagree to 6 = strongly agree), with higher scores indicating higher PsyCap. The scale demonstrates good reliability and validity, with a Cronbach's α of 0.819; in this study, the Cronbach's α for the scale was 0.948.

#### 2.3.5 Transition shock of newly graduated nurses scale (TSNGNS)

TS was assessed using the TSNGNS developed by Xue (38). The scale includes 27 items across four dimensions: physical (6 items), psychological (8 items), knowledge and skills (5 items), and sociocultural and developmental (8 items).

Items are rated on a 5-point Likert scale, with higher scores indicating greater TS. The scale demonstrates good reliability and validity, with a Cronbach's α of 0.918; in this study, the Cronbach's α for the scale was 0.969.

### 2.4 Data collection

Data were collected using Wenjuanxing, a secure online survey platform that provides encryption and access control. After obtaining approval from the head nurses of each hospital, the questionnaire was distributed anonymously. The first page explained the study purpose, data use, confidentiality, and voluntary participation. All items were mandatory, and participants completed the survey after providing informed consent electronically.

### 2.5 Ethical approval

This study was approved by the Ethics Committee of Hubei University of Arts and Science (No. 2025-33) and was conducted in accordance with the Declaration of Helsinki.

### 2.6 Statistical analysis

Data were analyzed using SPSS version 26.0 and Mplus version 8.3. LPA was conducted using Mplus 8.3 based on the scores of each dimension of the TSNGNS. Model fit indices included the Akaike information criterion (AIC), Bayesian information criterion (BIC), sample-size adjusted BIC (aBIC), entropy, the bootstrapped likelihood ratio test (BLRT), and the Lo–Mendell–Rubin likelihood ratio test (LMRT). Smaller values of AIC, BIC, and aBIC indicate better model fit, whereas an entropy value closer to 1 indicates higher classification accuracy. The LMRT and BLRT were used to compare K-class and K−1-class models; a *p*-value < 0.05 indicated that the K-class model was superior.

Categorical variables were presented as frequencies and percentages, and continuous variables as mean ± SD or median (P25, P75). Group differences were analyzed using chi-square tests, one-way ANOVA, or Kruskal–Wallis tests. Spearman's correlation was used to assess associations among variables. Mediation analysis was conducted using PROCESS (Model 6) with 5,000 bootstrap samples and 95% confidence intervals. A *p*-value < 0.05 was considered statistically significant.

## 3 Results

### 3.1 Common method bias

Harman's single-factor test identified four factors with eigenvalues greater than 1, and the first factor explained 34.44% of the total variance (< 40%) (39), suggesting that common method bias was not a serious concern.

### 3.2 Participant characteristics

A total of 438 NGRNs completed the survey. The mean age of the participants was 23.12 ± 1.17 years. Most participants were female (93.4%), worked in tertiary hospitals (78.1%), and were employed on a contract basis (87.7%). More than half held a bachelor's degree or higher (68.0%), and 32.9% worked in internal medicine departments. Additionally, 50.5% reported working more than 10 night shifts per month. Detailed characteristics are presented in Table 1.

**Table 1:** General characteristics of NGRNs (*n* = 438).

Characteristic	*N* (%)
Age, mean (SD)	23.12 ± 1.17
Hospital type, *n* (%)	
Secondary level A hospital	96, (21.9)
Tertiary-level grade A hospital	201, (45.9)
Provincial tertiary-level grade A hospital	141, (32.2)
Gender, *n* (%)	
Male	29, (6.6)
Female	409, (93.4)
Education, *n* (%)	
Specialist	140, (32.0)
Bachelor's degree	277, (63.2)
Master's degree	21, (4.8)
Employment type, *n* (%)	
Contract-based employment	384, (87.7)
Agency-based employment	34, (7.8)
Government-established posts	20, (4.6)
Department, *n* (%)	
Internal medicine	144, (32.9)
Surgery	85, (19.4)
Pediatrics	27, (6.2)
Obstetrics and gynecology	22, (5.0)
Emergency department	18, (4.1)
ICU	39, (8.9)
Operating room	21, (4.8)
Outpatient clinic	19, (4.3)
Other	63, (14.4)
Night shift frequency per month, *n* (%)	
< 5	195, (44.5)
5~10	22, (5.0)
>10	221, (50.5)

ICU, intensive care unit.

### 3.3 Correlations among TS, PsyCap, SVE, and PSC

**Figure 2:**
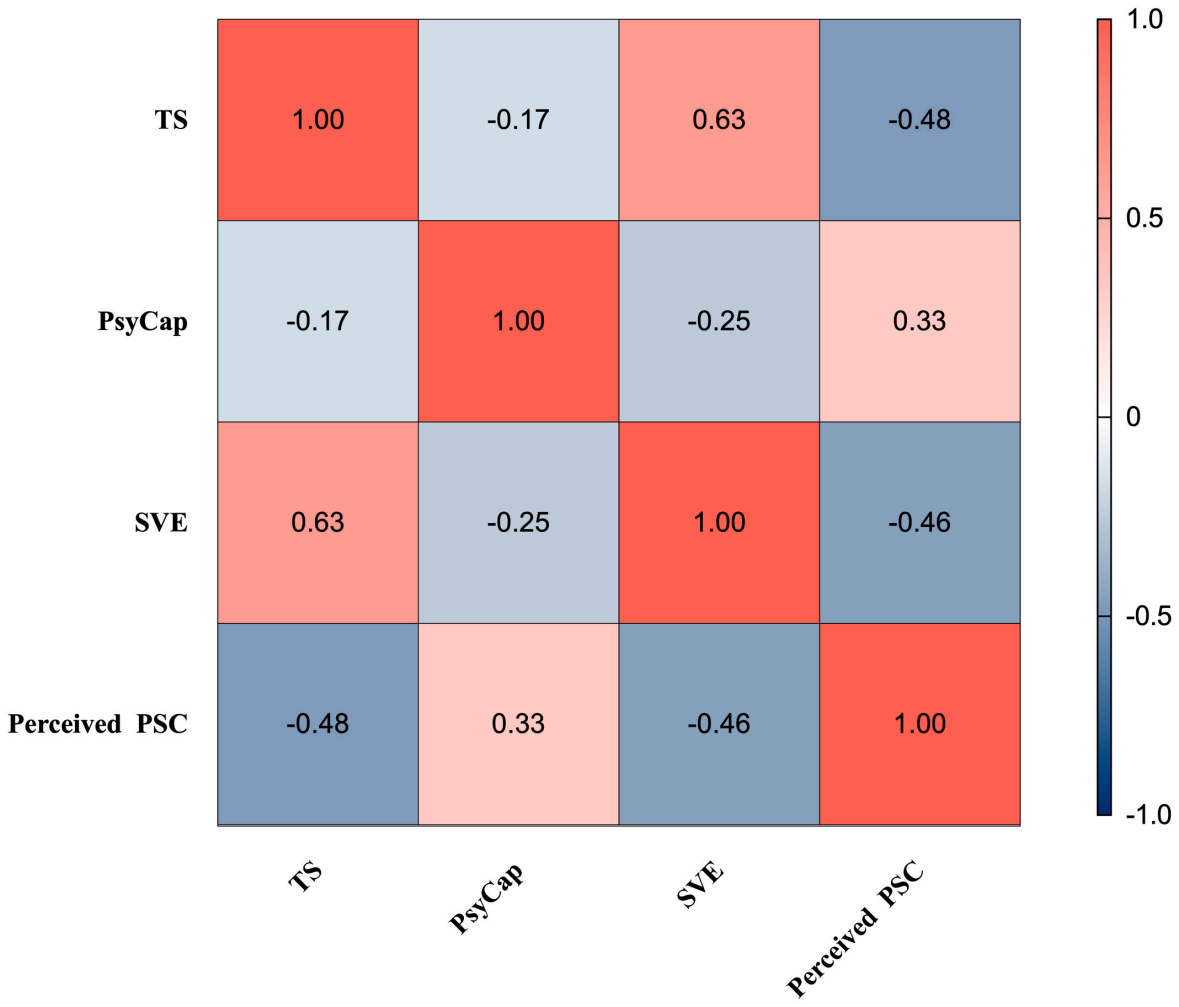
Descriptive and correlation analysis of scores for variables.

Spearman's correlation analysis showed that TS was negatively correlated with PSC (*r* = −0.464, *p* < 0.001) and PsyCap (*r* = −0.124, *p* < 0.01), and positively correlated with SVE (*r* = 0.656, *p* < 0.001). Detailed results are presented in Figure 2.

### 3.4 LPA of TS

Model fit indices are presented in Table 2. As the number of profiles increased, AIC, BIC, and aBIC values decreased progressively. When three profiles were specified, the decrease in these indices leveled off, and both LMRT and BLRT were significant (*p* < 0.001). Therefore, the three-profile solution was considered optimal and selected.

**Table 2:** Latent profile model fit indices for transition shock.

Profile	*K*	AIC	BIC	aBIC	Entropy	*p* values		Category ratio
						LMR	BLRT	
1	8	4,797.564	4,830.222	4,804.834				
2	13	4,080.394	4,133.463	4,092.207	0.846	< 0.001	< 0.001	0.443/0.557
3	18	3,725.952	3,799.432	3,742.309	0.890	< 0.001	< 0.001	0.480/0.365/0.155
4	23	3,560.315	3,654.206	3,581.216	0.882	0.006	< 0.001	0.361/0.139/0.354/0.146
5	28	3,514.493	3,628.795	3,539.937	0.821	0.024	< 0.001	0.130/0.228/0.235/0.130/0.277

**Figure 3:**
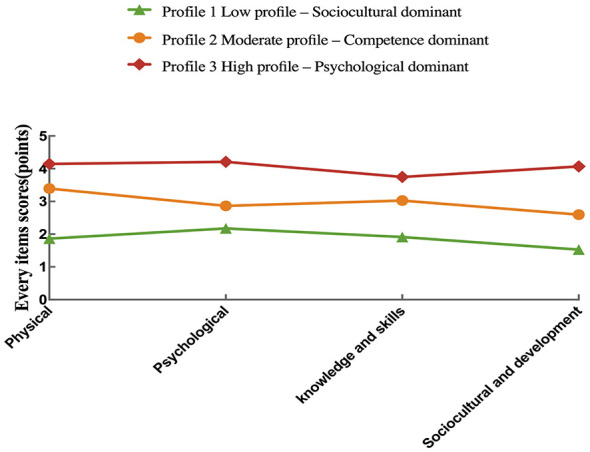
Distribution of three latent profiles of TS.

As shown in Figure 3, Profile 1 (low TS–social type) included 160 participants (36.5%) and was characterized by low scores across all dimensions. Profile 2 (moderate TS–competence type) included 210 participants (48.0%) and was characterized by moderate overall scores, with relatively higher scores in the knowledge and skills dimension. Profile 3 (high TS–psychological type) included 68 participants (15.5%) and was characterized by high scores across all dimensions, particularly in the psychological domain.

### 3.5 Demographic characteristics of NGRNs across the three profiles

Comparisons of demographic characteristics across the three latent profiles showed no significant differences in age, hospital type, gender, education level, employment type, department, or night shift frequency (all *p* > 0.05). These results indicate that the three profiles were generally comparable in terms of demographic characteristics. Detailed results are presented in Table 3.

**Table 3:** Differences in general characteristics of NGRNs across latent profiles.

Characteristic	Profile 1 (*n* = 160)	Profile 2 (*n* = 210)	Profile 3 (*n* = 68)		*p* value
Age, mean (SD)	23.17 ± 1.05	23.17 ± 1.21	23.43 ± 1.30	1.379	0.253
Hospital type, *n* (%)					
Secondary level A hospital	7, (24.1)	15, (51.8)	7, (24.1)	2.882	0.237
Tertiary-level grade A hospital	153, (37.4)	195, (47.7)	61, (14.9)		
Provincial tertiary-level grade A hospital					
Gender, *n* (%)	34, (35.4)	49, (51.0)	13, (13.6)	3.527	0.474
Male	76, (37.8)	88, (43.8)	37, (18.4)		
Female	50, (35.5)	73, (51.8)	18, (12.8)		
Education, *n* (%)					
Specialist	54, (38.5)	67, (47.9)	19, (13.6)	2.477	0.649
Bachelor's degree	101, (36.4)	132, (47.7)	44, (15.9)		
Master's degree	5, (23.8)	11, (52.4)	5, (23.8)		
Employment type, *n* (%)					
Contract-based employment	141, (36.7)	183, (47.7)	60, (15.6)	1.723	0.787
Agency-based employment	14, (41.2)	16, (47.1)	4, (11.7)		
Government-established posts	5, (25.0)	11, (55.0)	4, (20.0)		
Department, *n* (%)					
Internal medicine	62, (43.1)	56, (38.9)	26, (18.1)	16.468	0.421
Surgery	28, (32.9)	43, (50.6)	14, (16.5)		
Pediatrics	7, (25.9)	17, (63.0)	3, (11.1)		
Obstetrics and gynecology	8, (36.4)	14, (63.6)	0, (0.0)		
Emergency department	7, (38.9)	9, (50.0)	2, (11.1)		
ICU	15, (38.5)	18, (46.1)	6, (15.4)		
Operating room	8, (38.1)	9, (42.9)	4, (19.0)		
Outpatient clinic	6, (31.6)	12, (63.2)	1, (5.3)		
Other	19, (30.2)	32, (50.8)	12, (19.0)		
Night shift frequency per month, *n*, (%)					
< 5	72, (36.9)	92, (47.2)	31, (15.9)	1.989	0.738
5~10	83, (37.6)	105, (47.5)	33, (14.9)		
>10	5, (22.7)	13, (59.1)	4, (18.2)		

ICU, intensive care unit.

### 3.6 Differences in perceived PSC Scores by profile

Significant differences in overall PSC and all its dimensions were observed across the three profiles (*p* < 0.001). Compared with the Low TS—social type, the high TS—psychological type reported lower overall PSC scores and lower scores across all dimensions. The largest difference was observed in teamwork climate (Z = 50.240). Detailed results are shown in Table 4.

**Table 4:** LPA-based difference in PSC among NGRNs.

Variable	Profile 1 (*n* = 160)	Profile 2 (*n* = 210)	Profile 3 (*n* = 68)	F/Z	*p* value
Total score	92, (80.25, 108)	87, (74, 93)	71.5, (68.25, 86)	70.467	< 0.001
Teamwork climate	29, (28, 35)	28, (25, 31)	25, (23, 28)	50.240	< 0.001
Job satisfaction	24.42 ± 4.28	22.50 ± 4.10	19.40 ± 4.70	33.640	< 0.001
Stress recognition	10, (8, 13)	9, (7, 11)	8, (6, 9)	42.717	< 0.001
Unit safety climate	16.36 ± 3.00	14.90 ± 3.23	12.79 ± 3.18	31.737	< 0.001
Perception of management	11.65 ± 1.96	10.91 ± 2.07	9.97 ± 1.87	17.592	< 0.001

### 3.7 Mediating effects of PsyCap and SVE

Mediation analysis indicated that TS was negatively associated with PSC in the overall sample [β = −0.211, 95% CI (−0.277, −0.145)]. This relationship remained statistically significant across all profiles.

Regarding indirect effects, PsyCap was found to significantly mediate the relationship between TS and PSC in the low TS [β = −0.055, 95% CI (−0.141, −0.007)], moderate TS [β = −0.082, 95% CI (−0.160, −0.025)], and high TS groups (β = −0.130, 95% CI (−0.278, −0.013)].

SVE also demonstrated a significant mediating effect in the low TS [β = −0.069, 95% CI (−0.149, −0.008)] and moderate TS groups [β = −0.047, 95% CI (−0.114, −0.003)], but not in the high TS group [β = −0.043, 95% CI (−0.162, 0.051)].

The chain mediation pathway was significant in the low TS [β = −0.012, 95% CI (−0.033, −0.001)] and moderate TS groups [β = −0.018, 95% CI (−0.038, −0.004)], but not in the high TS group [β = −0.032, 95% CI (−0.148, 0.002)].

**Figure 4:**
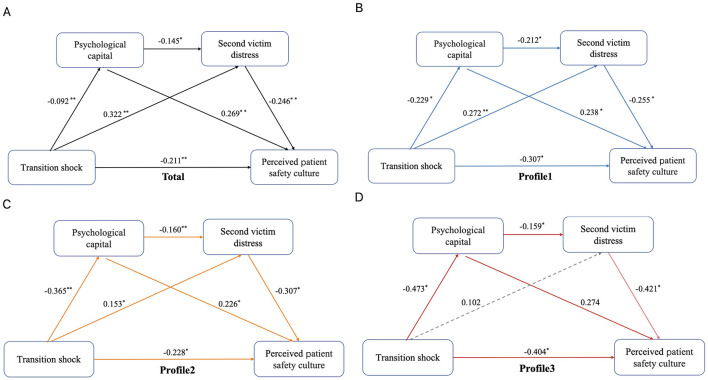
Overall and profile-specific mediation models of PsyCap and SVE in the association between TS and perceived PSC. **(A)** Overall sample: **(B)** Profile 1: Low transition shock: **(C)** Profile 2: Moderate transition shock: **(D)** Profile 3: High transition shock (*p* < 0.05; ^**^p < 0.01) Dashed lines indicate non-significant paths.

Overall, these findings support the presence of both independent and chain mediation effects. Detailed results are presented in Table 5, and Figure 4 illustrates the pathways.

**Table 5:** Mediation analysis of PsyCap and SVE based on LPA.

Types of effect	Model path	β	BootSE	95% CI		Effect ratio, (%)
				LLCI	ULCI	
Total effect		−0.318	0.028	−0.373	−0.264	
Direct effect	TS → PSC	−0.211	0.034	−0.277	−0.145	66.35%
Indirect effect	TS → PsyCap → PSC	−0.025	0.008	−0.043	−0.010	7.86%
	TS → SVE → PSC	−0.079	0.023	−0.126	−0.036	24.84%
	TS → Psy Cap → SVE → PSC	−0.003	0.002	−0.007	−0.001	0.95%
Profile1 total effect		−0.443	0.100	−0.641	−0.246	
Profile1 direct effect	Low TS → PSC	−0.307	0.103	−0.510	−0.104	69.30
Profile1 indirect effect	Low TS → PsyCap → PSC	−0.055	0.035	−0.141	−0.007	12.42
	Low TS → SVE → PSC	−0.069	0.037	−0.149	−0.008	15.57
	Low TS → Psy Cap → SVE → PSC	−0.012	0.008	−0.033	−0.001	2.71
Profile2 total effect		−0.375	0.099	−0.571	−0.180	
Profile2 direct effect	Moderate TS → PSC	−0.228	0.099	−0.423	−0.033	60.80%
Profile3 indirect effect	Moderate TS → PsyCap → PSC	−0.082	0.035	−0.160	−0.025	21.87%
	Moderate TS → SVE → PSC	−0.047	0.028	−0.114	−0.003	12.53%
	Moderate TS → Psy Cap → SVE → PSC	−0.018	0.009	−0.038	−0.004	4.80%
Profile3 total effect		−0.608	0.150	−0.907	−0.309	
Profile3 direct effect	High TS → PSC	−0.403	0.140	−0.684	−0.123	66.28%
Profile3 indirect effect	High TS → PsyCap → PSC	−0.130	0.069	−0.278	−0.013	21.38%
	High TS → SVE → PSC	−0.043	0.053	−0.162	0.051	7.07%
	High TS → Psy Cap → SVE → PSC	−0.032	0.041	−0.148	0.002	5.27%

TS, transition shock; PSC, patient safety culture; TS, transition shock; SVE, second victim experience.

## 4 Discussion

This study identified distinct profiles of TS among NGRNs using latent profile analysis and further examined the associations between these profiles and perceived PSC. The findings confirm that TS is not a uniform experience but exhibits significant heterogeneity, supporting Hypothesis 1. Furthermore, higher levels of TS were found to be associated with lower perceived PSC, which is consistent with Hypothesis 2. By integrating a person-centered approach with mediation analysis, this study provides a more nuanced understanding of the mechanisms through which transition shock influences PSC. These findings can be interpreted within the framework of COR theory, which posits that individuals strive to obtain, maintain, and protect valuable resources, whereas resource loss is more salient than resource gain. In this context, transition shock may be viewed as a significant source of resource depletion, and differences in perceived PSC across TS profiles may reflect varying degrees of resource loss and adaptation among NGRNs.

### 4.1 Heterogeneity of TS among NGRNs

This study identified three distinct TS profiles, with the “moderate TS–competence type” being the most prevalent profile, followed by the “low TS–social type,” while the “high TS–psychological type” accounted for a smaller but clinically important proportion. Notably, more than half of the participants experienced moderate to high levels of TS, suggesting that transition-related stress is widespread among NGRNs.

This finding may be interpreted within the context of the early career transition process. Newly graduated nurses are required to rapidly adapt to demanding clinical environments while simultaneously developing professional competence, managing complex patient care, and navigating interpersonal relationships (40). Frequent night shifts during the initial stages of employment can disrupt sleep patterns and increase physical fatigue (41). At the same time, due to limited clinical experience, new nurses often worry about their ability to cope with their roles and may experience additional psychological stress due to fear of making mistakes (42). Furthermore, they must quickly familiarize themselves with clinical procedures and enhance their clinical judgment and emergency response capabilities, thereby increasing pressures related to knowledge and skills acquisition (43, 44).

Importantly, the predominance of the “moderate TS–competence type” suggests that competence-related uncertainty may represent a central challenge during early career transition. Although NGRNs receive systematic theoretical training, they often lack confidence in applying knowledge in complex clinical situations, resulting in a mismatch between perceived competence and actual job demands. This imbalance may partly explain why competence-related stress appears more prominent than other dimensions.

In contrast, the “high TS–psychological type” reflects a more pervasive maladjustment, characterized by consistently elevated scores across multiple dimensions, particularly psychological distress. This suggests that once transition difficulties extend beyond competence-related challenges and begin to affect emotional regulation, the impact of TS becomes more profound. These individuals are more likely to experience sustained anxiety, tension, and frustration and may therefore require more intensive psychological support.

Interestingly, no significant demographic differences were observed across profiles, suggesting that TS heterogeneity may be driven more by internal factors, such as psychological resources and coping capacity, rather than by observable background characteristics. This finding is consistent with previous research emphasizing the role of individual psychological differences in shaping early career adaptation (16).

### 4.2 Differences in perceived PSC across TS profiles

Significant differences in perceived PSC were observed across the three TS profiles, with higher levels of TS associated with lower PSC, particularly in the dimension of teamwork climate. This finding provides further support for Hypothesis 2 and is consistent with prior studies indicating that work-related stress and inadequate adaptation negatively influence perceived PSC (45). From a theoretical perspective, high levels of TS may impair individuals' cognitive processing and emotional regulation, leading to more negative perceptions of workplace interactions and organizational support. When exposed to stress and uncertainty, NGRNs may become more focused on task completion and error prevention, while paying less attention to communication and collaboration, which are essential components of PSC. At the organizational level, lower levels of perceived PSC among high-TS nurses may undermine teamwork, communication, and error reporting, thereby hindering the development of a positive safety culture within healthcare organizations.

In addition, limited clinical experience and insufficient confidence may further hinder effective participation in team-based care (46). Under conditions of high TS, nurses may adopt more passive or avoidant behaviors, reducing their sense of belonging and perceived support within the team. These findings suggest that improving PSC requires not only organizational strategies but also attention to the psychological adaptation of NGRNs.

### 4.3 PsyCap plays a significant mediating role between TS and perceived PSC

The results indicate that PsyCap plays a consistent mediating role between TS and PSC across all profiles, supporting Hypothesis 3. As an important positive psychological resource, PsyCap enables individuals to maintain confidence, optimism, and resilience in the face of stress.

According to COR theory (27), individuals are motivated to maintain and protect their psychological resources, and stress occurs when these resources are threatened or depleted. Within this framework, TS may be conceptualized as a stressor that depletes psychological resources, leading to reductions in PsyCap. Previous studies have consistently demonstrated that higher levels of PsyCap are associated with better coping, lower stress, and more positive workplace perceptions among nurses (47).

Conversely, when TS is high, the depletion of PsyCap may reduce resilience and optimism, making individuals more vulnerable to stress and more likely to develop more negative perceptions of their work environment. This finding underscores the role of PsyCap as a key psychological mechanism linking transition shock to perceived PSC (16).

This study also found that SVE demonstrated a significant mediating effect in the overall sample, as well as in the low TS–social type and moderate TS–competence type, but did not reach statistical significance in the high TS–psychological type. This indicates that the role of SVE may vary across different levels of TS.

Following patient safety incidents, nurses often experience psychological reactions such as self-blame and anxiety, accompanied by doubts about their own capabilities, which may in turn affect both their professional wellbeing and their evaluation of the work environment (48). As the impact of TS increases, such negative experiences are more likely to accumulate and function as mediating mechanisms.

Interestingly, the chained mediation pathway was not statistically significant in the high TS group. Although the relatively small subgroup sample size may have reduced statistical power, this finding may also reflect a distinct psychological mechanism under conditions of extreme TS. According to COR theory, when NGRNs experience severe TS, their psychological resources may become substantially depleted, leading them to enter a resource-defensive state. This interpretation is supported by qualitative study (49) showing that NGRNs experience considerable psychological and emotional challenges during the transition process, particularly in high-intensity clinical environments, which may accelerate the depletion of internal resources. Under such circumstances, PsyCap may emerge as the primary factor influencing perceptions of PSC, whereas the mediating role of SVE becomes relatively attenuated. Previous studies (50) have shown that SVE distress is associated with adverse work outcomes, while internal psychological resources and organizational support can mitigate these negative effects. Therefore, once psychological resources fall below a critical threshold, the impact of TS on PSC may be transmitted predominantly through direct resource depletion rather than through subsequent adverse work experiences. This finding suggests that the mechanisms linking TS to PSC may differ across TS profiles.

Further analysis indicates that PsyCap and SVE form a chain mediation pathway in the overall sample as well as in the low and moderate TS groups. This suggests that the impact of transition shock is not driven by a single pathway, but rather by the combined effects of reduced psychological resources and increased exposure to negative professional experiences. Specifically, TS appears to influence PSC through a sequential process in which reduced PsyCap increases vulnerability to SVE. These findings highlight that both the depletion of positive psychological resources and the accumulation of negative professional experiences jointly contribute to variations in PSC.

### 4.4 Clinical implications

The findings of this study provide clear, evidence-based guidance for clinical practice and nursing management. Interventions aimed at reducing TS should extend beyond the initial orientation period and be sustained throughout the transition process (51). Given the heterogeneity of TS, profile-specific interventions may be more effective.

For nurses in the “moderate TS–competence type”, nursing managers should focus on facilitating role adaptation and strengthening professional competence. In addition to structured preceptorship programs, simulation-based training, and clinical decision-making exercises, hospitals should improve the transparency and realism of job descriptions and competency expectations, aligning pre-employment expectations with actual clinical responsibilities to reduce initial transition shock and frustration (52) Furthermore, competency-based recruitment and placement strategies can improve the fit between individual capabilities and job demands, reducing performance-related stress during the early transition period.

For nurses in the “high TS–psychological type”, protecting and restoring psychological resources should be prioritized. Healthcare organizations may establish peer-support programs, resilience and psychological capital development initiatives, and provide counseling services to help nurses recover from stress (53). Strengthening perceived organizational support through supportive leadership training, equitable resource distribution, and ensuring nurses feel valued and heard can further promote adaptation and PSC (54). At the organizational level, nursing leaders should foster open communication and psychological safety, provide visible managerial support, and adopt non-punitive approaches to patient safety incidents that emphasize learning and system improvement rather than individual blame (55) These measures may strengthen perceived organizational support and promote positive perceptions of PSC.

From the perspective of COR theory, these interventions may help prevent resource loss spirals and promote resource gain among NGRNs. By reducing transition shock, strengthening psychological capital, and providing timely organizational support, healthcare institutions may improve PSC, enhance workforce sustainability, and ultimately contribute to safer and higher-quality patient care.

### 4.5 Limitations

This study has several limitations. First, owing to the cross-sectional design, causal relationships among variables cannot be established. Second, although the overall sample size was adequate, the high TS–psychological type included only 68 NGRNs. Therefore, the statistical power for detecting relatively small indirect effects in subgroup mediation analyses may have been limited. Consequently, the non-significant mediation pathways observed in this subgroup should be interpreted with caution and warrant further verification in larger samples. Third, all variables were collected using self-reported questionnaires, which may introduce common method bias. Although Harman's single-factor test indicated that the first factor accounted for 34.44% of the variance, this method has limited sensitivity. Therefore, the findings should be interpreted cautiously. Future studies could employ longitudinal designs and multi-source data collection to further reduce potential common method bias and strengthen causal inference.

## 5 Conclusions

This study identified three distinct latent profiles of TS among NGRNs, confirming substantial heterogeneity. Higher levels of TS were associated with lower perceived PSC, and the mediating roles of PsyCap and SVE formed a chain pathway in specific subgroups. By integrating individual differences and psychological mechanisms, these findings clarify how TS influences PSC and provide empirical support for the applicability of COR theory in the professional transition context. Overall, the results offer important insights for nursing management and clinical practice to support NGRNs' adaptation, enhance psychological resources, and improve patient safety outcomes.

## Data Availability

The raw data supporting the conclusions of this article will be made available by the authors, without undue reservation.
